# Relationship between early proteinuria and long term outcome of kidney transplanted patients from different decades of donor age

**DOI:** 10.1186/s12882-019-1635-0

**Published:** 2019-12-02

**Authors:** Davide Diena, Maria Messina, Consuelo De Biase, Fabrizio Fop, Edoardo Scardino, Maura M. Rossetti, Antonella Barreca, Aldo Verri, Luigi Biancone

**Affiliations:** 1 Renal Transplant Center “A. Vercellone”, Nephrology, Dialysis and Renal Transplant Division, “Città della Salute e della Scienza Hospital”, Department of Medical Sciences, University of Turin, Corso Dogliotti14, 10126 Torino, Italy; 2 Division of Pathology, “Città della Salute e della Scienza Hospital”, Department of Medical Sciences, University of Turin, Turin, Italy; 3 Department of Vascular Surgery, “Città della Salute e della Scienza Hospital”, University of Turin, Turin, Italy

**Keywords:** Kidney transplantation, Elderly donors, Proteinuria, Long-term outcome, Estimated glomerular filtration rate

## Abstract

**Background:**

Proteinuria after kidney transplantation portends a worse graft survival. However the magnitude of proteinuria related to patient and graft survival and its correlation with donor and recipient characteristics are poorly explored.

**Methods:**

This study investigated the impact of post transplant proteinuria in the first year in 1127 kidney transplants analyzing the impact of different donor ages. Proteinuria cut off was set at 0.5 g/day.

**Results:**

Transplants with proteinuria > 0.5 g/day correlated with poor graft and patient outcome in all donor age groups. In addition, 6-month-1-year proteinuria increase was significantly associated with graft outcome, especially with donors > 60 years old (*p* <  0.05; Odd Ratio 1.8). 1-year graft function (eGFR < or ≥ 44 ml/min) had similar impact to proteinuria (≥ 0.5 g/day) on graft failure (Hazard Ratio 2.77 vs Hazard Ratio 2.46). Low-grade proteinuria (0.2–0.5 g/day) demonstrated a trend for worse graft survival with increasing donor age. Also in kidney-paired analysis proteinuria ≥0.5 effect was more significant with donors > 50 years old (Odd Ratio 2.3).

**Conclusions:**

Post-transplant proteinuria was increasingly harmful with older donor age. Proteinuria ≥0.5 g/day correlates with worse outcomes in all transplanted patients. Prognostic value of proteinuria and eGFR for graft and patient survival was comparable and these two variables remain significant risk factors even in a multivariate model that take into consideration the most important clinical variables (donor age, rejection, delayed graft function and cytomegalovirus viremia among others).

## Background

Over the past decades, increase of donor pool by using elderly donors has been largely adopted to reduce kidney transplant (KT) waiting list [1]. “Old for old strategy” allowed a better allocation of kidneys matching the life expectancy of organs and recipients [2], even if elderly kidneys have been shown to have a compromised renal reserve and more proclivity to nonspecific damages. Those conditions (in particular through ischemia-reperfusion damage and delayed graft function -DGF-) increase the immunologic risk of such organs either through enhanced immunogenicity or through compromised repair mechanisms [3]. Nevertheless, death censored graft survival does not change among donor decades if a correct allocation policy is performed [4].

Proteinuria is known to be an independent risk factor for cardiovascular disease and mortality in native kidneys as well as an indicator of renal damage and a predictor of allograft loss after kidney transplantation [5, 6]. Several studies in the past years speculated that the optimal timing for measuring proteinuria to detect ongoing damage and to adopt specific strategies to prevent its progression is between 3 months and one year post-KT [7–12].

Even if older donor age is mentioned as risk factor for development of post-KT proteinuria [8, 13], the effective impact of proteinuria in recipients of elderly donors is not clearly defined and studied. Moreover, in the great majority of the studies, mean donor age is far younger (on average 45 years) than in cohorts with prevalence of non-standard donors.

Halimi and coworkers showed a strong correlation of 1- and 3-month proteinuria (per every 0.1 g/day increase) with graft loss [7]**.** Amer and colleagues analyzed 1-year post KT proteinuria finding an Hazard Ratio (HR) for graft loss of 2.15 (CI 95% 0.68–6.8), associated with proteinuria between 150 and 500 mg/day and an HR of 5.11 (CI 95% 1.4–19.2) with higher level of proteinuria [9].

In a recent study Naesens et al. found a strong direct correlation between 1-year post KT proteinuria and graft loss irrespectively from graft histology, but only for proteinuria values higher than 1 g/day (HR 2.17) [6]. In the study by Cantarovich and coworkers, proteinuria > 0.5 g/d, at 3-month and 2-year post-KT was a significant prognostic marker of graft outcome. At 5-years, this significance was not observed [14].

To the best of our knowledge, only one available study is specifically focused on proteinuria impact on transplants from Extended Criteria Donors, although on a relatively small sample size and without comparison with standard donors [15].

Aim of this study was to analyze the impact of different degrees of 1-year proteinuria on patient and graft survival in kidney transplants from different donor ages and to evaluate the correlation between proteinuria and donor or recipient risk factors with graft loss. A secondary aim is to evaluate proteinuria as risk factor for kidney graft survival in multimodal models taking into account renal function and other main clinical variables.

## Methods

### Study design

We performed a retrospective observational cohort study including all deceased donor grafts performed at Turin University Renal Transplant Center “A. Vercellone” from January 2003 to December 2013. The study was submitted and approved by the local ethics committee (ethics committee of “Azienda Ospedaliera Universitaria Città della Salute e della Scienza di Torino”/University of Turin). To exclude confounding factors and homogenize study populations we excluded multi-organ grafts analyzing the remaining 1127 consecutive kidney transplants (KT).

Immunosuppressive regimen differed according to different transplantation eras and different populations. Briefly, the most adopted schedules were: induction with steroid boluses, rapidly tapered to 20 mg/day of oral prednisone and two doses of anti-CD25 antibody (Simulect®, Novartis, Basel, Switzerland). Subjects at high immunological risk (e.g. previous transplant lost for acute rejection, high PRA titer) were treated with antithymocyte globulin (Thymoglobuline®, Genzyme, Cambridge, USA). Maintenance therapy was generally based initially on a triple-drug protocol. Calcineurin inhibitor (CNI), either tacrolimus (80% of cases) or Cyclosporine, was associated to Mofetil Micofenolate (Cell Cept®, Roche, Basel, Switzerland)/Micophenolic Acid (Myfortic®, Novartis, Basel, Switzerland) or Azathioprine and prednisone. In KT from Extended Criteria Donors, CNI administration was, in most cases, delayed until serum creatinine reached 2.5 mg/dl to reduce the impact of nephrotoxicity, as we previously published^15^. CNI levels were targeted on the basis of patients’ characteristics, KT time and transplant eras; for the vast majority of patients, target plasmatic tacrolimus levels were: 10–15 ng/ml in the first two months, 8–10 ng/ml up to the sixth month, 5–8 ng/ml up to the first two years. Steroid was tapered to 5 mg/day within 45–60 days and subsequently withdrawn in selected patients. Micofenolate dose reduction and discontinuation were performed when appropriate after hospital discharge. Mammalian target of Rapamycin-inhibitors (mTORi), combined or not with low doses CNI, were used in selected cases (anamnestic/active malignancies, CNI intolerance, biopsy-proven severe CNI toxicity) with a switch generally after the sixth post-transplant month.

The recipients’ follow-up was performed with scheduled clinical visits for the entire follow-up and hospital admissions when major complications occurred. Data were collected from patients’ individual charts: creatinine and proteinuria (in 24-h urine collections) were assessed at discharge, at 3, 6 months and at 1, 2 and 5 years after transplantation. Renal allograft function (eGFR) was estimated by Chronic Kidney Disease Epidemiology Collaboration (CKD-EPI) equation. Pre-transplant donor biopsies were performed on the basis of a multidimensional assessment including macroscopic appearance, renal function, donor comorbidities and echographic characteristics [4]. In detail: in the great majority of cases biopsy is usually not performed in donors < 50 years and always performed in donors older than 70 years. For donors with age 50–70 several characteristics are taken into consideration including donor cerebrovascular cause of death in the absence of vascular malformation, echographic parameters (renal cortex thickness, renal longitudinal diameter, longitudinal diameter discrepancy between the two kidneys), donor hypertension, donor diabetes, donor active smoking, donor proteinuria > 100 mg/dl at urinalysis.

Post-transplant renal biopsies were performed for cause (mainly when serum Creatinine increased ≥20% of baseline value or with proteinuria > of 0.5–1 g/day). A single group of trained pathologists, through the whole study period, analyzed all pre-transplant and post-transplant kidney biopsies. Follow-up ended on November 2017. Karpinsky score was used to define suitability for single or dual KT or refusal of organs.

**Fig. 1:**
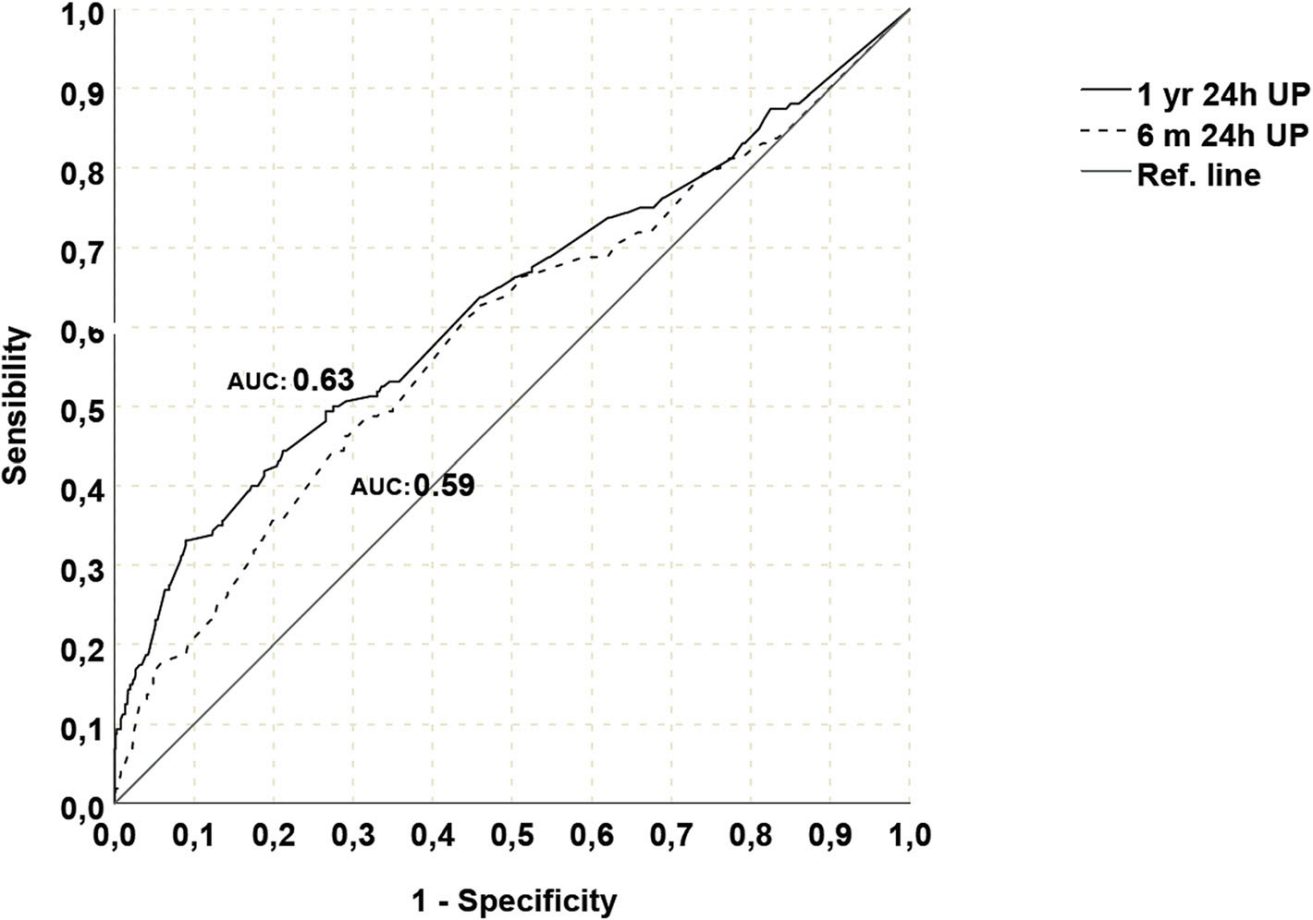
ROC curves for association between 6-month and 1-year post kidney Transplantation proteinuria and death censored graft survival, *M = month, yr = year, AUC = Area Under the curve, UP = urinary protein*

Patients were divided in 3 groups according to donor age: group A (< 50 years), group B (50–69 years), group C ≥ 70 years.. The outcomes were analyzed for patients with at least 1 year of follow-up according to 1-year post-KT extent. To eliminate confounding factors, as native kidneys proteinuria, we mainly explored proteinuria values after the sixth post-KT month; when comparing 6-month and 1-year proteinuria (1-year PTO), the latter showed a better correlation with death censored graft survival (DCGS) with an AUC 0.64 vs 0.59 (Fig. 1).

**Fig. 2:**
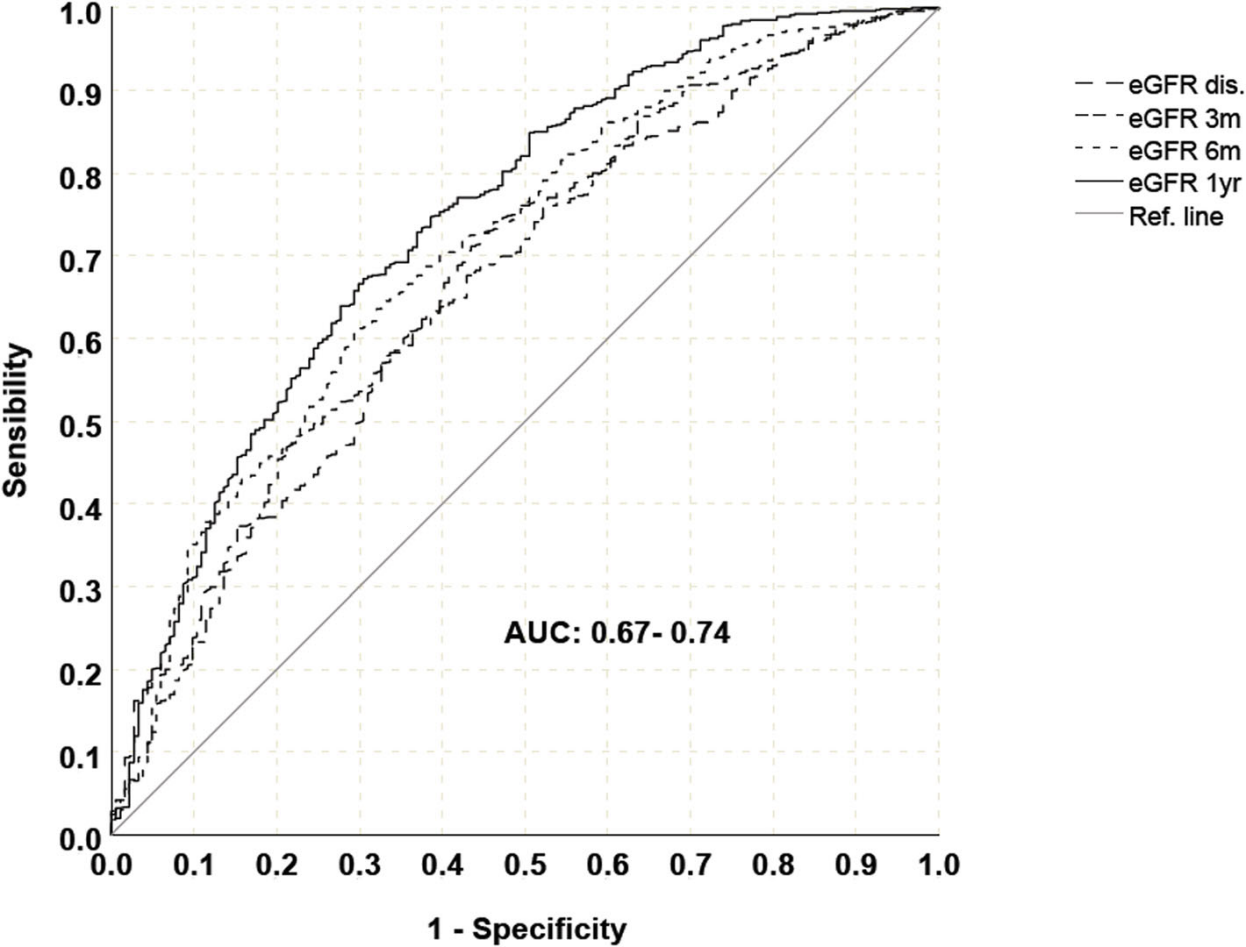
ROC curves for association between eGFR at different time points after kidney transplantation and death censored graft survival, *Egfr = estimated glomerular filtration rate, dis = discharge, m = month, y = year, AUC = Area Under the Curve*

Regarding 1-year PTO we used a cut-off based on clinical considerations and previous literature data (0.5 g/day). On the basis of median value of proteinuria in our cohort we further analyze low-grade proteinuria (between 0.2 g/day and 0.5 g/day). Similarly,eGFR value at 1-year post-KT showed the best correlation with DCGS (AUC 0.74) (Fig. 2) and its median value in the whole population was 44.24 ml/min (25st–75st percentiles, 32.92–58.77 ml/min). Main outcomes were death censored graft survival (DCGS) and patient survival. Graft function and the occurrence of relevant post-KT complications were also analyzed.

A further paired kidney analysis was performed considering only cases where both kidneys from the same deceased donor were transplanted at our institution (370 recipients, 185 pairs); primary outcomes were evaluated in this subset as in general cohort.

### Ethics and consent to participate

The study was submitted and approved by the local ethics committee (ethics committee of “Azienda Ospedaliera Universitaria città della Salute e della Scienza di Torino”/University of Turin) and was performed in adherence with the last version of the Helsinki Declaration. All patients allowed to have their clinical data collected for study purposes by written consent. The clinical and research activities being reported are consistent with the Principles of the Declaration of Istanbul as outlined in the **“**Declaration of Istanbul on Organ Trafficking and Transplant Tourism”.

### Statistical methods

Discrete data were described as percentages and analyzed with Pearson’s *X*^2^ or, for small samples, with Fisher’s exact test. The distribution of continuous variables was analyzed with Kolmogorov-Smirnov test. Continuous variables were described as mean ± standard deviation when normal and median with 25°-75° percentile when non-normal distributed. Mann-Whitney, Kruskal-Wallis, t-test or variance analysis with Bonferroni post hoc test were used when appropriate to analyze difference between groups.

Receiving operating characteristic (ROC) curve and under the curve area (UCA) were used to illustrate the diagnostic ability of parameters chosen. We used UCA to determine the proteinuria time point with the strong association with DCGS.

Cumulative graft and patient survival were analyzed by Kaplan-Meier (KM) curves.

We fitted univariate model for main clinically chosen covariates (1- year proteinuria, donor age, recipient age, eGFR, DGF, rejection, 1 year Cytomegalovirus (CMV) infection) and we identified predictors as significant at level α = 0.05 (log rank test). Then we fitted a multivariate model with univariate predictors using stepwise with *p*-value < 0.05 as entry criterion and p-value > 0.10 as removal criterion.

All statistical analyses were performed using Spss (IBM SPSS Statistics, vers. 25.0.0). Significance level for all tests was set at α <  0.05.

## Results

Our analysis included 1127 kidney recipients, transplanted at Turin University Renal Transplant Center “A. Vercellone” between January 2003 and December 2013. The selected population was classified by donor age: group A (less than 50 years old) including 339 patients, group B (50–69 years old) including 496 patients and group C (more than 70 years old, with a maximum age of 88 years) including 292 patients. Main donor, recipient and transplant-associated characteristics are shown in Table 1. Mean follow-up was 8.21 years (25th–75th percentiles: 5.38–11.43 years).

**Table 1:** Donor, Recipient and Transplant baseline characteristics by donor age < 50 years between 50 and 69 years and ≥ 70 years

	All patients (1127 pts)	Donors < 50 yrs. (339 pts)	Donors 50–69 yrs. (496 pts)	Donors ≥70 yrs. (292 pts)	*P* value
1A - Donor characteristics					
M/F (%)	578/549 (51/49)	215/124 (63/37)	229/267 (46/54)	134/158 (46/54)	*< 0,01*
eGFR CG (ml/min)	99,38	126,89 ± 40,65	94,68 ± 34,83	76,78 ± 28,22	*< 0,01*
eGFR CKD-EPI(ml/min)	83,65	103,51 ± 30,39	85,79 ± 25,38	79,7 ± 22,03	*< 0,01*
Hypertension (%)	511 (49)	66 (21)	259 (57)	186 (68)	*< 0,01*
Diabetes mellitus (%)	78 (8)	9 (3)	49 (12)	20 (8)	*< 0,01*
Cerebrovascular cause of death (%)	747 (72)	159 (52)	370 (80)	218 (81)	*< 0,01*
1B - Recipient characteristics					
M/F (%)	721/406 (64/36)	217/122 (64/36)	305/191 (61/39)	199/93 (68/32)	*0,18*
Mean age (Yrs)	59.17 ± 9.43	43,99 ± 10,69	55,31 ± 9,66	62,73 ± 7,93	*< 0,01*
1st Tx/ More than 1 Tx (%)	972/155 (87/13)	277/62 (82/18)	427/69 (86/14)	268/24 (92/8)	*< 0,01*
SKT/DKT (%)	1082/45 (96/4)	339/0 (100/0)	481/15 (97/3)	262/30 (90/10)	*< 0,01*
HD/PD (%)	873/ 308 (79/28)	279/73 (86/22)	390/136 (80/28)	204/99 (71/35)	*< 0,01*
Pretransplant DM 1 or 2/type 2 (%)	95/79 (10,2/7)	19/14 (6/4)	48/35 (10/7)	16/30 (16/11)	*< 0,01*
Pretransplat Hypertension (%)	939 (86)	267 (81)	420 (88)	252 (91)	*< 0,01*
Pretransplant Cardiopathy (%)	358 (32)	90 (26)	164(33)	104 (36)	*< 0,01*
Pretransplant HCV POS (%)	91 (8)	26 (8)	44 (9)	21 (8)	*0,78*
1C - Transplant characteristics					
HLA A/B/DR MM (0–2/3–4/5–6) %	48/46/6	32/62/6	34/57/9	42/54/4	0,27
PRA zero (CDC) at transplantation %	66,3	63	63	75,4	0,13
Cold ischemia time (hours)	16,16 ± 5,22	15,89 ± 5,37	17,80 ± 4,98	18,25 ± 4,64	0,03
DGF (%)	298 (28)	74 (23)	135 (29)	89 (32)	*0,04*
**Induction Therapy**					*< 0,01*
ATG (%)	21 (2)	3 (1)	3 (1)	9 (3)	
Basiliximab (%)	1080 (98)	319 (98)	479 (98)	282 (99)	
**Mantaining Therapy**					*< 0,01*
Tacrolimus (%)	848 (79)	286 (87)	360 (77)	202 (73)	
Cyclosporine (%)	181 (17)	32 (10)	95 (20)	54 (20)	
mTORi (%)	83 (8)	27 (11)	31 (7)	25 (9)	
mTORi at 1 yr(%)	169 (15)	77 (23)	59 (12)	33 (11)	*< 0,01*
ACE/ARB (%)	368 (33)	190 (56)	117 (24)	61 (21)	*< 0,01*
**End f-up Mantaining Therapy (%)**					*< 0,01*
Tacrolimus (%)	839 (78)	271 (83)	374 (79)	194 (69)	
Cyclosporine (%)	133 (12)	27 (8)	64 (13)	42 (15)	
mTORi (%)	255 (24)	56 (17)	124 (26)	73 (26)	

eGFR = estimated Glomerular filtration rate; CG = Cockroft-Gault formula; CKD-EPI = Chronic Kidney Disease Epidemiology Collaboration; SKT = Single Kidney Transplantation; DKT = Dual Kidney Transplantation; PD = Peritoneal Dialysis; HD = Haemodialysis; DM = Diabetes Mellitus; HCV = Hepatitis C virus; HLA = Human Leucocyte Antigens; MM = Mismatch; PRA = Panel Reactive Antibodies; CDC = Cell Dependent Cytotoxicity; ATG = anti-thymocite globulin; mTORi = mammalian target of rapamycin inhibitors; ACE = angyotensin converting enzyme; ARB = Angiotensin Receptor Blockers; DGF = delayed graft function

Assuming 0.5 g/day as proteinuria cut-off, the association of 1-year PTO with DCGS and graft survival was present for all donor age classes (Table 2); the impact of proteinuria on patient survival was noted only for younger donors. Donor age increased the magnitude of proteinuria impact: DCGS of patients with donor age ≥ 70 years and higher 1-year proteinuria was only 29.7% versus 72.3% in recipients of kidneys from younger donors with the same proteinuria (*p* = 0.03).

**Table 2:** Patient, graft and death censored 10-year graft survival by different 1-year proteinuria and by different donor age classes

10-years survival %	1-year pto < 0,5 g/day	1-year pto ≥ 0,5 g/day	P value
All donor age classes			
Patient	87	81,3	0,02
Graft	76.4	44.4	< 0,01
DCGS	85.6	49.7	< 0,01
Donor < 50 years			
Patient	96,9	79,6	< 0,01
Graft	90.6	65.9	< 0,01
DCGS	93.6	72.3	< 0,01
Donor 50–69 years			
Patient	86,9	87,9	0,67
Graft	74.9	43.1	< 0,01
DCGS	84.4	48.2	< 0.01
Donor ≥70 years			
Patient	71,2	71,6	0,44
Graft	56.2	25.9	< 0,01
DCGS	75,2	29.7	< 0,01

DCGS = death censored graft survival; srv = survival; KT = kidney transplantation; pto = proteinuria

**Fig. 3:**
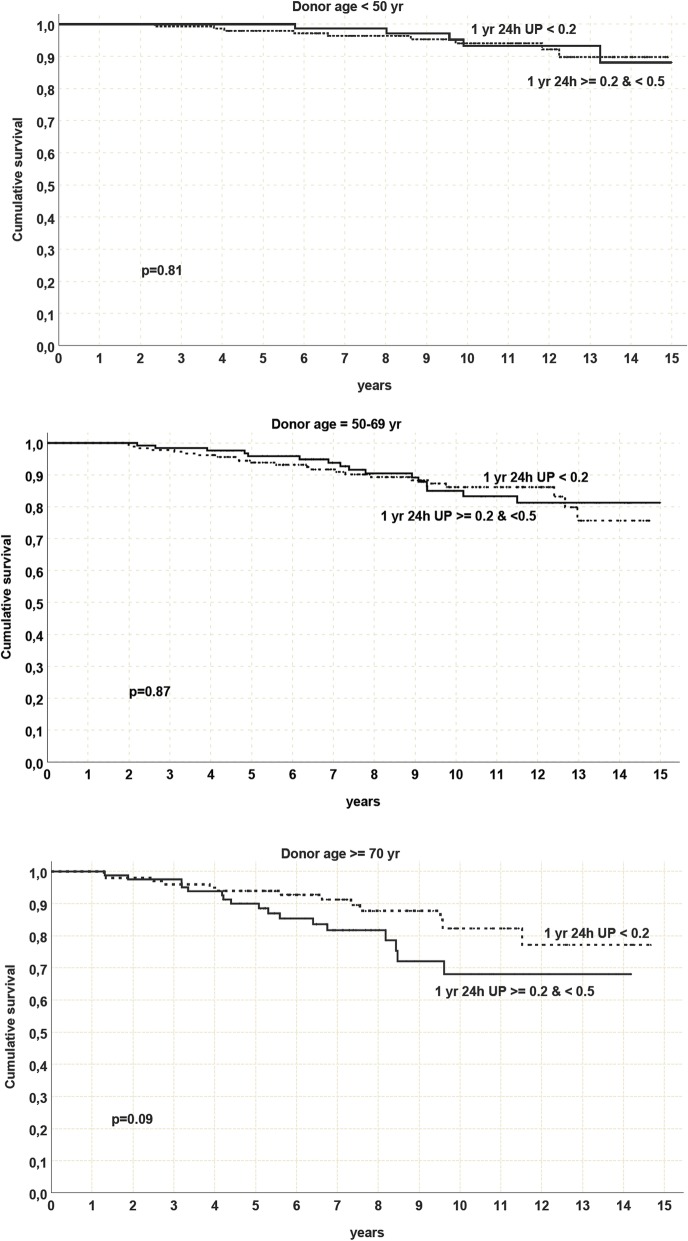
Death censored graft survival in patient with 1-year proteinuria 0.2–0.5 g/day compared with proteinuria < 0.2 g/day in the whole population and by different donor age, *Yr = year, UP = urinary protein*

As we noticed that median value of proteinuria in our population was nearly 0.2 g/day, we explored the impact of low grade proteinuria (0.2–0.5 g/day) compared with proteinuria < 0.2 g/day in the whole cohort and in different donor ages. In the low grade proteinuria group univariate analysis did not show any significant association of 1-year PTO with patient and graft survival and DCGS at any donor age. Yet, a definite (not significant) trend was evident for donors ≥70 years, regarding graft and DCGS (DCGS 82.3% with 1-year proteinuria < 0.2 g/day vs 65.3% with 1- year proteinuria 0.2–0.5 g/day; *p* = 0.09) Fig. 3.

In order to investigate whether other donor factors could be related with post-KT proteinuria, Karpinsky score was evaluated when pre-implantation biopsies were available (*n* = 567), together with various factors (hypertension, diabetes, cause of death, serostatus for C hepatitis).

In particular, regarding histology, we analyzed the distribution of total Karpinsky score in recipients of single KT and in different donor age groups finding a significant difference (*p* <  0.05; data not shown). Moreover we analyzed distribution of total Karpinsky score in different one-year proteinuria groups (< or ≥ 0.5 g/day) without finding significant differences (*p* = 0.59; data not shown), while a higher glomerulosclerosis score showed a good correlation with a higher 1-year proteinuria (*p* = 0.04). Nevertheless, total Karpinsky score as well as glomerulosclerosis score were not associated with DCGS differences.

We also performed another analysis splitting population under study by donor age and by one-year proteinuria but again we found no correlation between total Karpinsky score and DCGS in any of group analyzed.

We further took into consideration, short-term variation of proteinuria between 6-month and 1-year post-KT (6mo-1 yr proteinuria): in 44.0% of patients proteinuria increased between these 2 time points while in 56.0% it remained stable or decreased. Median positive variation of proteinuria was 0.12 g/day (19,8% of patients had an increase of proteinuria ≥0.1 g/day) while median negative variation was 0.05 g/die (22.4% of patients had a decrease of proteinuria ≥0.1 g/day). Positive/negative variation values were comparable for different donor age groups.

**Fig. 4:**
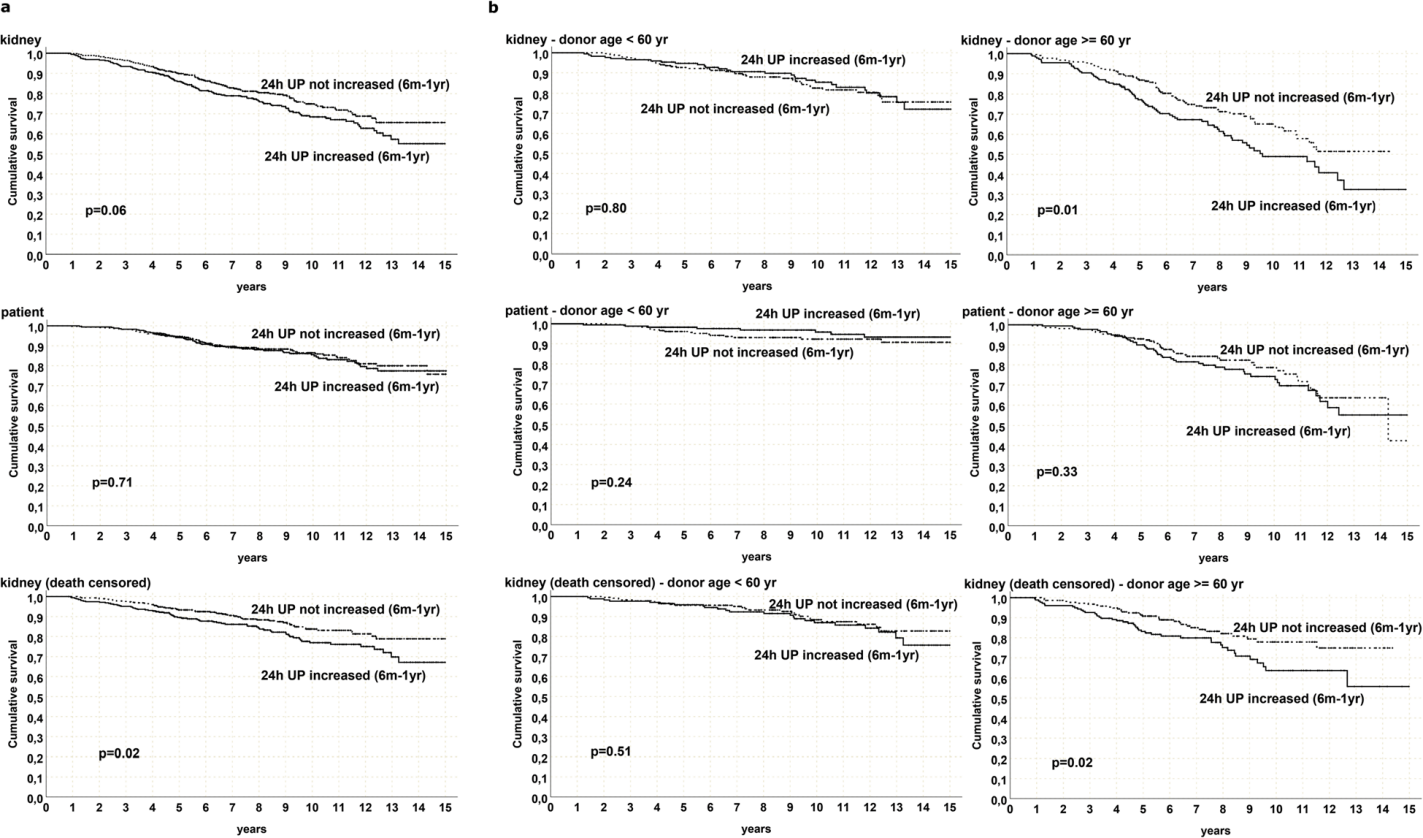
**a** Patient, graft and death censored graft survival by decreasing/stable or increasing proteinuria between 6-months and 1-year after kidney transplantation, in the whole population, **b** Patient, graft and death censored graft survival by decreasing/stable or increasing proteinuria between 6-months and 1-year after kidney transplantation divided by donor ages: < 60 and ≥ 60 years old, *M = month, yr = year, UP = urinary protein*

6mo-1 yr proteinuria increase was not associated with patient survival at any donor age (*p* = 0.71) (Fig. 4). On the contrary, any increase of proteinuria between these time points was associated with poor graft survival and DCGS (AUC 0.6; OR 1.8 – CI 95% 1.2–2.5).

Again, this correlation was stronger when graft and DCGS were considered in recipients of elderly donors (Fig. 4). The different time to graft failure between donor age groups, respectively 6 years (CI 3–9 years) in group A vs 3 years (CI 1–6 years) in group C, can be considered an additional data in favor of the association between donor age and 6mo-1 yr proteinuria positive variation. Angiotensin Converting Enzyme inhibitors/ Angiotensin II Receptor Blockers (ACEi/ARB) therapy did not influence 6mo-1 yr proteinuria variations.

Including only subjects with 6mo-1 yr proteinuria stable or increasing (*n* = 558) we identified 0.1 g/day as 6mo-1 yr proteinuria cut-off with the best association with DCGS (UCA 0.68).

Patients with increase in 6mo-1 yr proteinuria higher than 0.1 g/day had lower DCGS if compared with patients with lower increase (10-year graft survival 69.9% vs 90.2%; *p* <  0.01). That significant difference was confirmed irrespectively of donor age.

To test the role of a potential confounding factor, the same analysis was performed excluding patients mTor inhibitors treated at one year post transplantation (*n* = 159). In the remaining patients, proteinuria impact (≥ 0.5 g/day) was not associated with significant variation in DCGS in the different age classes in univariate analysis (data not shown).

Early adverse events (during first post KT year) were evaluated in the different proteinuria subpopulations (Table 3). As expected, 1-year post-KT proteinuria > 0.5 g/day was associated with new onset diabetes (NODAT), glomerulonephritis and rejection. Notably, also transplant urologic complications and Cytomegalovirus (CMV) viremia had an association with proteinuria. Biopsies were significantly more frequent in patients with higher 1 year proteinuria.

**Table 3:** Complications in the first year post-KT in the different group by 1-year proteinuria < 0,5 g/day and ≥ 0,5 g/day

	All patients	1 year PTO < 0,5 g/day	1 year PTO ≥ 0,5 g/day	P value
*Post transplant complications*				
DGF %	25,4	24,4	30,7	< 0,05
Transplant Urologic Complications %	30	28,3	38	< 0,05
Ureteral fistula %	3,8	3,1	7,0	< 0,05
Ureteral stenosis %	6,4	5,2	12,7	< 0,05
Transplant Vascular Complications %	15,5	14,6	19,7	0,12
Vascular fistula %	4,7	4,4	6,5	0,36
Renal artery stenosis %	9,4	9,0	11,5	0,18
Overall infection %	37,2	35,9	45,2	0,07
CMV infections %	24,2	22,3	34,4	< 0,05
Ischemic Cardiopathy %	7,9	8,3	5,7	0,33
NODAT %	21,2	19,7	29,3	0,01
Extrarenal Major Vascular Complications%*	2,6	2,5	3,2	0,68
Hypertension %	88	85	96	0,07
Rejection %	6,1	5,8	12,1	< 0,05
Biopsies %	26,9	23,1	55,4	< 0,05
Total Glomerulonephritis/Recurrent glomerulonephritis %	9,3/2,5	7,3/1,7	15,9/6,3	< 0,05

DGF = delayed graft function; CMV = cytomegalovirus; NODAT = new onset diabetes after transplantation. Biopsies are all performed for cause

*Including stroke, arterial stenosis and thrombosis

**Fig. 5:**
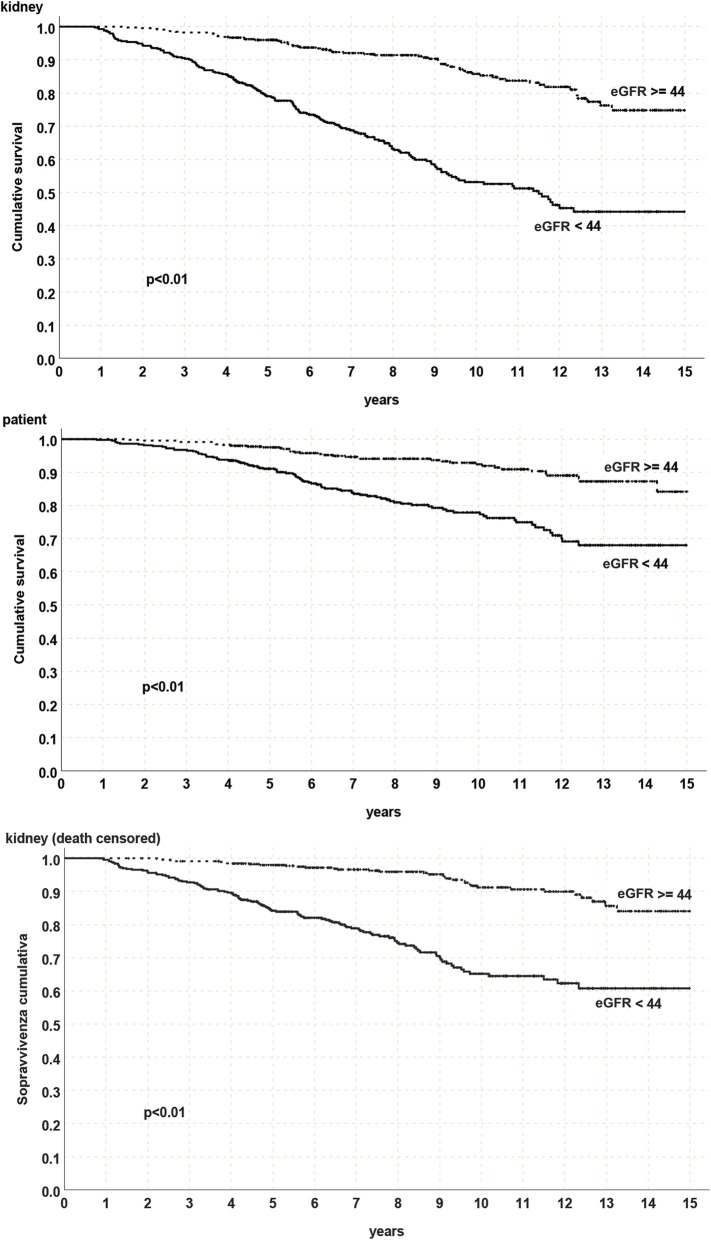
Patient, graft and death censored graft survival with 1-year eGFR (CKD-EPI) ≥ or < of 44 ml/min, *eGFR = estimated glomerular filtration rate*

To compare proteinuria and creatinine impact on graft survival, 1-year eGFR was also evaluated. 1-year eGFR was strongly associated to patient, graft and DCGS (91.2% with eGFR ≥44 ml/min versus 65.2% with eGFR < 44 ml/min; p <  0.01) (Fig. 5).

Finally, in order to select the main predictors of DCGS, a multivariate model was created considering the most relevant clinical variables: 1-year proteinuria, donor age, recipient age, eGFR, DGF, rejection, 1-year CMV infection.

In this multivariate analysis (Table 4) 1-year PTO ≥ 0.5 g/day, donor age ≥ 70 years, 1-year eGFR < 44 ml/min and the onset of CMV viremia in the first year were independently associated with DCGS. Rejection was a significant variable only when considered in the entire follow-up (not when 1st year rejections were considered).

**Table 4:** Cox model for variables affecting graft outcome (death censored). Reference population is represented by recipients of donors < 50 years

	P value	Hazard Ratio(Confidence interval 95%)
1-year proteinuria 0,2–0,5 g/day	0.63	0,90 (0.58–1,39)
1-year proteinuria ≥0,5 g/day	< 0.01	2,77 (1,81-4,22)
1-year eGFR (CKD-EPI) < 44 ml/min	< 0.01	2,46 (1,57-3,84)
Donor Age 50–69 years	0,36	1,27 (0,75-2,14)
Donor Age ≥ 70 years	0,029	1,85 (1.06–3,21)
1-year CMV viremia	< 0.01	2105 (1,47-3,00)
Rejection in the entire follow-up	< 0.01	2,57 (1,79-3,69)

CKD-EPI = Chronic Kidney Disease Epidemiology Collaboration, eGFR = estimated glomerular filtration rate, CMV = cytomegalovirus

Besides, on the basis of the heavy influence of donor age ≥ 70 years on DCGS, we attempted a kidney paired study to eliminate donor characteristics contribution to the analysis and evaluate other potentially influencing factors. One hundred-eighty five kidney pairs, with both kidneys from the same donor transplanted in our Center, were selected and, among them, 43 couples with 1-year proteinuria discrepancy (one graft with 1-year proteinuria ≥0.5 g/day and the paired one with proteinuria < 0.5 g/day) were analyzed.

**Fig. 6:**
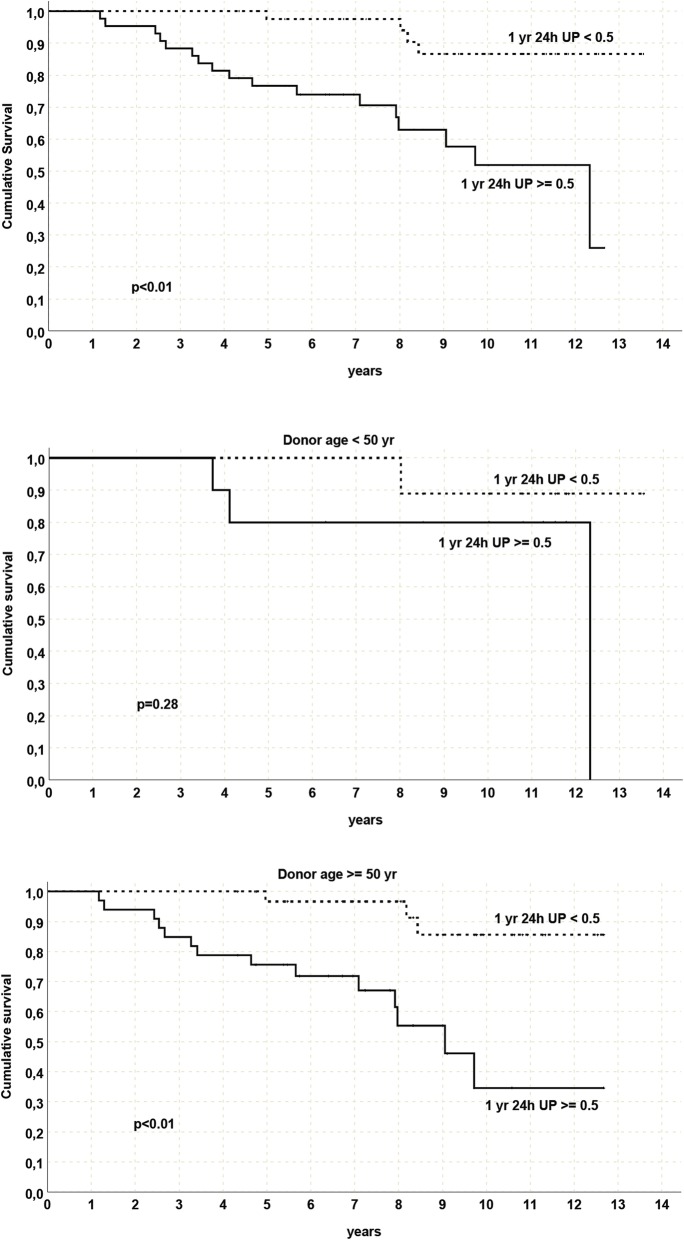
Patient, Graft and Death censored graft survival analysis in kidney pairs with proteinuria < 0,5 g/day or ≥ 0,5 g/day in the whole population and divided by donor age < 50 years or ≥ 50 years, *Yr = year, UP = urinary protein*

Also In this case a great impact on DCGS was found: DCGS was respectively 86.6% for recipients with proteinuria < 0.5 g/day and 51.9% for the twin kidney with proteinuria > 0.5 g/day; *p* <  0.01. This impact was not relevant in recipient of donors < 50 years while was greater with donors ≥50 years Fig. 6. Comparison of post-KT complications between the two groups confirmed a statistically higher rate of rejection and glomerulonephritis and greater number of biopsies in the group with 1-year PTO ≥ 0.5 g/day. No significant variation was noted between the groups for the other analyzed variables. (Table 5).

**Table 5:** Complications in the first year post-KT in the pairs with different 1-year proteinuria < 0,5 g/day and ≥ 0,5 g/day

	Kidney pairs (86 pts)	Kidney twin with 1 year PTO < 0,5 g/L (43 pts)	Kidney twin with 1 year PTO ≥ 0,5 g/L (43 pts)	P value
Post-Transplant Complications in 1st post-KT year				
DGF %	24 (28)	8 (19)	16 (38)	*0,03*
Transplant Urologic Complications %	48 (56)	23 (53)	25 (58)	*0,41*
Ureteral fistula %	3 (3)	1 (2)	2 (5)	*0,5*
Ureteral stenosis %	7 (8)	1 (2)	6 (14)	*0,05*
Transplant Vascular Complications %	19 (22)	11 (26)	8 (19)	*0,3*
Vascular fistula %	8 (9)	5 (12)	3 (7)	*0,36*
Renal artery stenosis %	9 (10)	4 (9)	5 (12)	*0,5*
Overall infections %	66 (78)	31 (74)	35 (81)	*0,28*
CMV infections	29 (34)	13 (30)	16 (37)	*0,32*
Ischemic Cardiopathy	7 (8)	5 (12)	2 (5)	*0,22*
NODAT	22 (26)	9 (21)	13 (30)	*0,32*
Extrarenal Major Vascular Complications*	18 (21)	10(23)	8(19)	*0,60*
Hypertension	27 (96)	12 (44)	15 (56)	*0,57*
Rejection	7 (8)	0 (0)	7 (16)	*< 0,01*
Biopsies (%)	51 (65)	18 (49)	33 (80)	*< 0,01*
Glomerulonephritis (%)	7 (8)	1 (2)	6 (14)	*0,05*

DGF = delayed graft function; CMV = cytomegalovirus; NODAT = new onset diabetes after transplantation. All Biopsies are performed for cause

*Including stroke, arterial stenosis and thrombosis

## Discussion

For the general population, the risk of adverse outcomes (mortality, progression to end-stage renal disease) increases with higher levels of albuminuria so that it was included in 2012 Kidney Disease Improving Global Outcomes guidelines as key marker for chronic kidney disease (CKD) [16].

Numerous studies in patients with diabetic and non-diabetic renal diseases confirm that marked albuminuria (> 300 mg/day) is associated with a faster rate of CKD progression. On the contrary, moderate-level albuminuria (150–300 mg/day) is not a reliable surrogate marker for CKD progression in intervention clinical trials because reduction of albuminuria can be linked to both improving and worsening CKD progression [17, 18].

The deleterious impact of proteinuria, at relatively early post-KT time, on long-term outcome was clearly demonstrated in several previous studies [6, 7, 9, 14]. Nevertheless, available studies differed in definition of post-KT harmful proteinuria as well as in post-KT considered time points. That uncertainty was reflected also by available guidelines for transplant management that suggest performing allograft biopsy when there is new onset proteinuria or unexplained proteinuria 3.0 g/mg creatinine or 3.0 g/24 h, with a low level of evidence [19].

Evaluation of risk factors related to post-KT proteinuria was attempted in several studies finding a multitude of different donor-related, recipient related or transplant related factors including, among others, delayed graft function, greater body mass index at transplant, older donor age, greater HLA mismatch, tacrolimus use and antihypertensive use [13, 20].

Beyond the causes, however, the intrinsic risk of developing proteinuria at a relatively early stage (first post-KT year) should be addressed as a key risk factor for transplant outcome [21].

Our study demonstrated that occurrence of proteinuria,(≥ 0.5 g/day), at the first post-transplant year, was significantly linked to graft survival and patient survival in the whole population under study. For the first time in literature, to the best of our knowledge, the impact of proteinuria on KTs from different donor age classes was analyzed, demonstrating a synergic effect of proteinuria ≥0.5 g/day and donor age ≥ 70 years on DCGS.

Very low grade proteinuria (between 0.2 and 0.5 g/day) was not related to outcome. Nevertheless, we demonstrated an association (a trend, not reaching significance) between DCGS and low-grade PTO (≥ 0.2 <  0.5 g/day) only in kidneys from donors older than 70 years old. (Fig. 3).

Older donor kidneys seemed more sensitive to proteinuric injuries in comparison with younger ones with very relevant differences of DCGS in different donor age classes with same proteinuria. This is also highlighted by the fact that any variation of proteinuria between 6 months and 1 year post-KT portends a worse graft outcome when donor age was ≥60 years (Fig. 4).

With ageing, kidney undergoes through processes that lead to reduced functional reserve and also to a lower renal reserve response to higher functional requests (e.g. protein load) due to a reduced capacity of renal autoregulation [22–25]. These functional changes, that could be also encountered in diabetic patients, were found to be associated with or precede microalbuminuria and glomerular lesions [26, 27]. Indeed, proteinuria is not considered to be a “normal” physiological process of aging also in cases when the development of a persistent proteinuria increases with age, due to the higher prevalence of diabetes, hypertension and paraproteinemias in the elderly [28]. Therefore it could be speculated that the aforementioned process could be amplified in kidneys retrieved from elderly donors, which became more prone to several insults leading to compensatory hyperfiltration of glomeruli that survive reperfusion injury, rejection, and drug toxicity, and,in ultimate analysis, to a faster progression of renal damage. In this context, proteinuria is, at the same time, marker of damage progression and established loss of function, as witnessed by concordance between proteinuria grade and renal function in transplanted patients.

Predisposition to proteinuria development in our population appeared to be certainly related to both donor and recipient characteristics (donor age, pre-transplant diabetes, glomerular Karpinsky score) but, as it is demonstrated by paired kidney analysis, early post-KT events (acute rejection, CMV infections, new onset diabetes after transplantation and urological complications such as ureteral stenosis) contribute to determine kidney fate and prognosis. As shown in Table 3, rejections, number of biopsies, NODAT and glomerulonephritis were associated with higher 1-year PTO, surprisingly with similar distribution between donor age groups (data not shown); overall infections and CMV viremia as well as vascular and urological complications were, on the contrary, significantly more frequent in older donor population (data not shown). These data could be explained as a consequence of the indication for decreasing immunosuppressive therapy in case of infection and of the lower quality of older donor tissues in comparison with the younger donor kidneys [29, 30]. Notably, we found a strong correlation between overall infection and rejection rates, especially when donor age was > 50 years. Therefore, even if rejection risk seems to be similar in all donor ages, susceptibility to external factors (such as infections or urological/vascular complications) plays a major role in older donor populations.

It is well known that kidney allograft function in a stable condition (usually between 3 months and 1 year post KT) is an important predictor of graft failure [31, 32]. One possible explanation is that, as in chronic kidney disease, lower kidney function is often associated with other cardiovascular risk factors (e.g. hypertension, dyslipidemia and smoke) predisposing to cardiovascular disease and mortality [33]. In our study (Table 4) we showed that 1-year proteinuria ≥0.5 g/day (HR 2.77) is comparable to CKD-EPI < 44 ml/min (HR 2.46) in predicting graft failure by multivariate analysis. In this context, donor age ≥ 70 years would make this association even worse.

Among the other clinical variables, CMV viremia post-transplant resulted as an independent predictor of DCGS in Cox multivariate analysis (HR 2.1), as mentioned in previous studies [34, 35]. As for rejection, when we consider rejection as an event in the entire follow-up, its role is comparable to the one of the other main risk factors (HR respectively 2.5 vs 2.1 and 2.4) (Table 4). This is not found for early rejections (1st year rejections) possibly because their role is somehow downsized in a context of a population of older donors and recipients in which other factors are probably more relevant.

Need for surrogate endpoints to enhance trial feasibility has been outlined by a recent review where proteinuria has been defined as a prognostic biomarker [36].

In the current scenario, the majority of available organs are represented by “suboptimal” donors (formerly known as ECD or with a Kidney Donor Profile Index greater than 85% according to the recent USA definition). Our study demonstrated prognostic significance of proteinuria, in particular with this kind of donors.

The link among proteinuria, donor age and subsequent higher proteinuria-mediated damage in older donors is an important issue of our study. Several mechanisms were advocated for proteinuria mediated tissue damage such as intratubular complement activation [37, 38], intratubular protein overload [39–41], radical oxygen damage induced by tubular reabsorption of iron carrying proteins such as transferrin [42]. Older donor kidneys may potentially be more sensitive to such mechanisms, even with a lower grade of proteinuria.

As mentioned in previous studies and confirmed by our data, the evidence suggesting a benefit for ACEi/ARB use in transplant recipients is still lacking. They showed that the use of these agents was often associated with a significant reduction in proteinuria and eGFR without a concurrent improvement of patient or allograft survival as it does in non-transplant settings such as in diabetic nephropathy [43, 44]. This is also confirmed in a recent randomized controlled trial in which ramipril compared with placebo did not lead to a significant reduction in doubling of serum creatinine, end-stage renal disease, or death in kidney transplant recipients with proteinuria. These data would not support widespread use of this drugs to obtain clinical improvement in transplanted patients [45].

It is well known that Mtor-inhibitors may induce proteinuria by targeting glomerular podocytes [46]. This is confirmed also in our cohort. However, excluding patients with mTor maintenance therapy in the first year (16%), the impact of proteinuria on outcome was confirmed.

Our study has some strenghts and some limitations. Strenghts of the study are related to homogeneity of the population, characterized by a wide range of data coming from over a thousand of KTs performed with the same team of surgeons, nephrologists and pathologists. Patients were centrally followed in the long term with all data recorded in patients’ charts.

Another strength, in our opinion, is the validation of prognostic impact of proteinuria in a subset of paired kidneys, thus limiting undetermined donor-derived confounding factors [47].

We acknowledge that a limitation of this study is the absence of protocol graft biopsies for center policy; however, this limitation reduces its importance when we consider that proteinuria impact was shown by some authors to be independent from the underlying renal allograft histology [6].

Other limitations are: absence of routinely donor specific antibody evaluation in the first year, which was available only in a minority of patients, so that we did not evaluate our population under this aspect; moreover lack of qualitative differentiation of urinary protein, considering that tubular or glomerular proteinuria could have different impact on graft outcome, as underlined in previous studies [11, 48, 49].

## Conclusions

If it is unlikely that a single noninvasive biomarker will yield a high predictive performance for graft loss, it is however auspicable a correlation of post-KT proteinuria trajectories with clinical events to guide clinical measures. Clinicians often do not know how to handle early low-grade proteinuria given the fact that it is often considered as aspecific, possibly due to native kidneys residual function (in the early period). Our study clearly evidence that proteinuria is always deleterious to transplant outcome even at early follow up time point. Based on our results we suggest that, in the context of elderly donors and in the absence of acknowledged effective pharmacological tools, when other causes of proteinuria are excluded (e.g. cardiovascular diseases, infections, metabolic comorbidities), close monitoring of proteinuria should be repeatedly performed. Dealing with immunologic “low-risk” patients, where utility of protocol biopsy is still debated, the presence of proteinuria in the first year at a relatively low extent (0.5 g/day), even in the absence of donor specific antibodies, should suggest a careful evaluation of patients leading to for-cause biopsy. Utility of protocol biopsy in patients with non-standard donor, even in the absence of risk factors, should be ascertained by further studies.

## Data Availability

All relevant data are enclosed in manuscript or in table and figures. The datasets used and/or analysed during the current study are available from the corresponding author on reasonable request.

## Abbreviations

6mo-1 yr: From 6 months to one year
ACEi: Angiotensin converting enzyme inhibitors
ARB: Angiotensin II receptor blockers
CI: Confidence interval
CKD: Chronic kidney disease
CKD-EPI: Chronic Kidney Disease Epidemiology Collaboration
CMV: Cytomegalovirus
CNI: Calcineurin inhibitors
DGF: Delayed graft function.
DCGS: Death censored graft survival
ECD: Extended criteria donors
eGFR: Estimated glomerular filtration rate
HR: Hazard ratio
KM: Kaplan-Meier
KT: Kidney transplantation
mTORi: mammalian target of Rapamycin
NODAT: New onset after transplantation diabetes
OR: Odds ratio
ROC: Receiver operating characteristics
UCA: Under the curve area
